# Primary care physicians, acupuncture and chiropractic clinicians, and chronic pain patients: a qualitative analysis of communication and care coordination patterns

**DOI:** 10.1186/s12906-016-1005-4

**Published:** 2016-01-25

**Authors:** Lauren S. Penney, Cheryl Ritenbaugh, Charles Elder, Jennifer Schneider, Richard A. Deyo, Lynn L. DeBar

**Affiliations:** 1 South Texas Veterans Health Care System, 7400 Merton Minter Blvd, San Antonio, TX 78229 USA; 2 The University of Arizona-Department of Family and Community Medicine, 1450 N Cherry Ave, Tucson, AZ 85719 USA; 3 Kaiser Permanente-Center for Health Research, 3800 N. Interstate Avenue, Portland, OR 97227-1098 USA; 4 Oregon Health and Science University-Department of Family Medicine, Oregon Health and Science University, Mail Code FM, 3181 SW Sam Jackson Park Road, Portland, OR 97239 USA

**Keywords:** Chronic musculoskeletal pain, Complementary and alternative medicine, Managed care system, Interprofessional communication, Chronic care, Acupuncture, Chiropractic

## Abstract

**Background:**

A variety of people, with multiple perspectives, make up the system comprising chronic musculoskeletal pain (CMP) treatment. While there are frequently problems in communication and coordination of care within conventional health systems, more opportunities for communicative disruptions seem possible when providers use different explanatory models and are not within the same health management system. We sought to describe the communication system surrounding the management of chronic pain from the perspectives of allopathic providers, acupuncture and chiropractor (A/C) providers, and CMP patients.

**Methods:**

We collected qualitative data from CMP patients (*n* = 90) and primary care physicians (PCPs) (*n* = 25) in a managed care system, and community acupuncture and chiropractic care providers (*n* = 14) who received high levels of referrals from the system, in the context of a longitudinal study of CMP patients’ experience.

**Results:**

Multiple points of divergence and communicative barriers were identified among the main stakeholders in the system. Those that were most frequently mentioned included issues surrounding the referral process (requesting, approving) and lack of consistent information flow back to providers that impairs overall management of patient care. We found that because of these problems, CMP patients were frequently tasked and sometimes overwhelmed with integrating and coordinating their own care, with little help from the system.

**Conclusions:**

Patients, PCPs, and A/C providers desire more communication; thus systems need to be created to facilitate more open communication which could positively benefit patient outcomes.

## Background

While estimates of chronic musculoskeletal pain (CMP) prevalence vary, CMP is both common and costly [1–4], and difficult to manage with conventional treatments. Indeed, CMP symptoms are among the top five reasons that patients visit clinics and emergency departments [4, 5]. People with CMP frequently utilize both conventional and complementary and alternative medicine (CAM) therapies [6]. Acupuncture and chiropractic (A/C) care are considered the most highly accepted by physician groups [7, 8] with the best evidence to support their use [9–12].

Although progress is being made, poor integration of care remains a challenge across the US health care system [13]. As more insurers offer alternative treatment benefits [14] and as more physicians support the use of CAM treatments for pain management [15], additional potential coordination difficulties arise. Research suggests little communication occurs directly between allopathic providers and their CAM counterparts [16, 17], making this an important place to study communication within a patient care management network.

Thus far, most research on coordination within this care network in non-integrative medicine settings has focused the perspectives of patients [18–20], allopathic providers [21], CAM providers [22], or care dyads, such as patients and allopathic providers [23–25] or allopathic and CAM providers [26–29]. This work has identified areas that frequently inhibit better care coordination, such as lack of disclosure of CAM use by patients [18, 24], poor interprofessional communication [22, 28], and providers working from different explanatory models and utilizing distinct sets of jargon [21]. However, researchers have generally not examined how treatment coordination is simultaneously viewed by patients, allopathic providers, and CAM providers. With few exceptions [30, 31], they have also not looked at these groups within the contexts of systems in which providers are working for the same insurer system but are not co-located or within an integrative medicine program.

This paper presents qualitative data collected as part of a large mixed methods study of the impact of acupuncture and chiropractic as implemented in usual care of CMP [32]. The goal of the qualitative data analysis presented here is to describe the communication system surrounding the management of chronic pain from the perspectives of allopathic providers, A/C providers, and CMP patients. We identify points of divergence and communicative barriers among the main stakeholders in the system. Rather than only pointing to problems within any one of the dyadic relationships, we discern how communication systems occur within a managed care program, and where opportunities exist for more fluid care coordination.

## Methods

### Design

This paper draws on data gathered during the second phase of a multi-phase, mixed-method study to evaluate the outcomes of real-world acupuncture and chiropractic (A/C) services for CMP (see [32] for a full description of the study). Qualitative methods were employed during phase two to gain a better understanding of the characteristics of A/C services received by users and the decision-making processes patients and allopathic providers used when choosing A/C services. This information was used in the design of the third phase’s prospective cohort study. Additionally, during analysis, two consistent themes manifested across participant groups: communication and access challenges, and use of opioid drugs. This paper is a result of an exploration of the former theme as it emerged within our data. Systemic communication and access issues were not a focus of the study, rather a complication we uncovered in the use of A/C because of qualitative data gathering.

### Setting

Kaiser Permanente Northwest is an HMO providing medical care to approximately 530,000 members in Oregon and Washington. Nearly all members have a chiropractic care benefit, and most (with the exception of Medicare patients) have an acupuncture benefit. These two clinical services represent the overwhelming majority of complementary and integrative care provided to members, and are thus the focus of this analysis. Kaiser Permanente Northwest contracts with Complementary Health Plans, which is a network of acupuncturists, chiropractors, and other clinicians, to provide clinical acupuncture and chiropractic care. All credentialing and quality of care monitoring for acupuncturists and chiropractors is performed by the Complementary Health Plan network. Patients with musculoskeletal pain can be referred by an HMO primary care or specialty physician to a Complementary Health Plan acupuncturists or chiropractor for a limited number of visits when clinically indicated. Referrals are first vetted by the Kaiser Permanente Northwest referral office for appropriateness, and after approval, the patient can select and appoint with a Complementary Health Plan clinician.

### Participants

This paper draws on data gathered through interviews and focus groups with managed care system CMP plan members who had and had not used acupuncture and/or chiropractic therapies (*n* = 90), allopathic PCPs with low to high referral rates to A/C care (*n* = 25), and contracted community A/C providers who treated a high volume of managed care CMP patients (*n* = 14). More detailed discussion of the overall project methods can be found in the design paper [32]; the Phase 2 methods described there closely match the methods used here. All interviews and focus groups were audio-recorded and transcribed for analysis and quality assurance. The Institutional Review Board of Kaiser Permanente Northwest approved all procedures. Consent forms were reviewed and signed by participants at the beginning of focus groups or interviews. All interviews were conducted by trained interview staff from the Kaiser Permanente Northwest Center for Health Research, which has a long track record of careful and responsive research within the health plan.

A total of 90 CMP health plan members participated in either a focus group (*n* = 80) or an individual interview (*n* = 10). Participants were identified from among those who endorsed a willingness to participate and consented to outreach at the end of a large-scale survey of Kaiser Permanente Northwest members that queried information about patterns of chiropractic and acupuncture utilization. (For complete results of that survey, see [33]) The survey provided information on participants’ use of acupuncture and chiropractic that allowed for stratification of focus group composition (managed care plan referral or self-pay; acupuncture or chiropractic). Our 11 focus groups were composed of the following: we held two focus groups for patients with an HMO referral to acupuncture, two for patients with an HMO referral to chiropractic care, two for patients who had received other acupuncture (e.g., self-referred and out-of-plan care), two for patients who had other chiropractic care (e.g., self-referred and out-of-plan care), and three for comparable CMP patients who have not received either acupuncture or chiropractic care. Each focus group contained between six and 10 individuals.

Overall, letters were sent to 480 eligible survey respondents; 63 actively refused, 90 participated in 11 focus group sessions (*n* = 80) or interviews (*n* = 10), and the remainder did not return messages or were not pursued once focus groups were filled. Because Portland has few individuals of minority race/ethnicity, and because of concerns that their experiences might differ in unknown ways, individuals who further endorsed a minority race on the survey were selected from each of the focus group pools to be specifically invited for individual interviews using the same interview guide as the focus groups. Individual interviews allowed greater flexibility in timing and location of interviews to enhance participation. Thirty-seven (of the 480) letters were mailed to these individuals, and 10 participants were interviewed. Demographic data for patients were collected as part of that survey [33]. Patient participants were 67.7 years of age on average and 70 % were female. The racial/ethnic breakdown was 76 % white, 8 % African American, 2 % Native American, 6 % other, and 8 % unknown/refused to state. As noted above, ethnic minorities were specifically oversampled to increase their representation in the study.

We also conducted 25 PCP interviews, distributed nearly evenly among PCPs (internal and family medicine) who were high, medium and low for acupuncture and/or chiropractic referrals (four to five PCPs/cell) according to plan referral records for both types of services. Level of referral was determined by comparing individual PCP referral frequency to their HMO colleagues from January 1, 2008 to June 30, 2010. High referrers were defined as those at the 80^th^ to 100^th^ percentile of referrals, with at least 15 patients referred to A/C. Moderate referrers were at the 40^th^ to 60^th^ percentile of referrals, with five to 10 referrals to A/C. Low referrers were those at the 0 to 20^th^ percentile, with two to three patient referrals to A/C.

We sent invitation emails to 86 PCPs; 13 actively declined, and the remainder were in some stage of establishing contact when the study cells were filled.

We similarly recruited acupuncturists and chiropractors, who saw a high volume of CMP patients from the health plan based on health plan referral records, from community settings in Oregon and Southwest Washington. A recruitment list for A/C providers was generated in two ways. Primarily we asked Complementary Health Plan administrators to identify a list of providers who received a high volume of referrals for HMO patients. Additionally, several PCPs who participated in the study suggested A/Cs they were aware of from their patients’ experiences. Interviews were completed with eight acupuncturists (out of 27 recruited) and six chiropractors (out of 21 recruited).

## Analysis

Qualitative coding was conducted using Atlas.ti software. Using the interview guide as a basis, an initial code book was created with five broad thematic areas and related sub-codes. For example, under the thematic code Decision Making and Referral Journey were child codes such as Beliefs about CAM and Referral Process. Codes were further refined after initial coding was completed and emerging themes identified. An informal reliability coding process was used to ensure conceptual clarity. Coder reliability was determined through duplicate coding of one out of every six interviews and focus groups. The coders compared how each transcript was coded and discussed discrepancies. In some cases these conversations led to refinement of code definitions in the code book and, in a few cases, the recoding of transcripts.

For this paper, we analyzed codes related to communication between patients and providers, communication between CAM and allopathic providers, the referral process, and treatment barriers. Although acupuncture and chiropractic are quite different therapies, we have combined them here because (1) referrals for both utilize exactly the same procedures in the health plan, and these are the only CAM therapies with frequent referrals for pain; (2) PCPs rarely make clear distinctions between them; and (3) the issues raised in the focus groups and patient interviews regarding referral and communication with the health plan were virtually identical. For the purposes of this paper, the similarities in situations (i.e. they were all dealing with the same communication issues, under similar referral guidelines) vastly outweigh the minor differences between them. For a similar reason in our analysis we have combined all PCP’s responses, regardless of referral level, because we found they talked about communication issues in the same way due to working under the same system conditions.

## Results

### Acupuncture and chiropractic referral

**Fig. 1:**
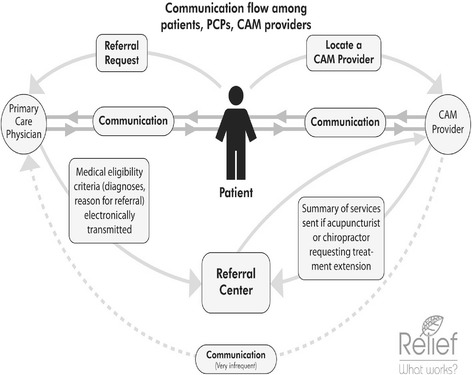
Communication flow among patients, PCPs, CAM providers. This illustrates the complex flow of communications among patients with chronic musculoskeletal pain, primary care providers (PCPs), and complementary and alternative medicine (CAM) providers within and outside of a managed care system. The CAM providers are acupuncturists and chiropractors

Frequently, a patient is referred to A/C after he or she makes a personal request. Figure 1, developed from the interview data, provides a schematic of the communication pathways. PCPs generally did not initiate discussions about A/C with their CMP patients. Patients who asked for referral often had previous or current experience with CAM modalities, knew someone who had success with those treatments, or had heard they were services available through the insurer. However, not all CMP patients had knowledge of or exposure to CAM, or knew that they could receive that care under their insurance benefits.

Both PCPs and patients described physicians as having a variety of responses to patient requests for A/C: from immediate assent, to recommendations to try more conventional therapies first, to denial. Some PCPs reported they might also selectively refer patients who had previous positive A/C experience. According to PCPs, they would usually only outright deny a request for referral if the patient’s medical condition contraindicated acupuncture or chiropractic treatment according to the benefit guidelines. However, some admitted that they would sometimes submit referrals even when they knew they would be denied because they wanted to appease the patient, were anxious to help the patient, and/or did not have time to personally deny the patient.

Occasionally patients had A/C proposed by their PCPs. Physicians were selective about which patients they recommended to A/C; it was not a possibility they opened to all their patients. They might discuss these therapies with patients with conditions they believed would be most responsive (e.g. did not want to take opioids), or patients who seemed more open to or had previous experience with CAM treatments. Many expressed the belief that A/C care was largely successful because of placebo, and would be less effective if the patient was not open to or believing in them. When physicians held such views, referrals to A/C were often deemed to be unproductive if the patient was perceived to not believe in CAM . However, in focus groups, patients who were naïve to CAM said they would be open to trying A/C if their physicians, who they trusted, suggested it. Notably, many PCPs stated they did not know enough about A/C, nor about the practitioners who were treating the patients, to feel qualified to make decisions about referrals.

For patients granted a referral, the next step was choosing from a list of A/C providers. Almost all of the Health Maintenance Organization (HMO) providers reported that, because of lack of familiarity, they were not able to refer patients to any particular provider. The process of selecting a provider could be daunting for patients, particularly those who were used to the HMO system in which they faced few similar provider choices. Patients made selections based, for example, on word of mouth, office location, and random selection from the list of available providers. In some cases, patients used friends, family, and personal experiences to guide them. Less frequently, PCPs might have a community provider they could recommend, often based on personal or other patients’ experiences. When lacking other guidance, many patients selected a provider based on ease of access (e.g., proximity to home), provider credentials (e.g., medical degree), or arbitrarily chose from the list.

Physicians expressed concern over not being able to provide patients more guidance, seemed anxious about the lack of oversight, and questioned the quality controls for ensuring patient treatment. PCPs, patients, acupuncturists and chiropractors all described variability among A/C provider practices and quality, which made choosing from a list of providers with no other context somewhat risky. Given the lack of feedback in the communication system to PCPs, there were not opportunities for them to learn about differences among various A/C treatments and providers, or to be better informed when referring patients to the community for A/C treatment.

### Communication among PCPs, acupuncturists, and chiropractors

Direct communication channels between PCPs and A/C providers were almost non-existent (see Fig. 1). Table 1 provides illustrative quotes from physicians and A/C providers on this issue. Because A/C providers were external to the HMO system, there were no systematic or institutionalized ways for sharing information or even knowing the names of other providers. A/C providers submitted some treatment paperwork to the HMO’s referral center, but this information was not routinely shared with PCPs. Lack of time and interest on the part of PCPs, as well as A/C provider uncertainty about receptivity of allopathic physicians to interaction, were additional barriers. Patients were relied upon to communicate with their providers and share information; however this was neither consistent nor complete.

**Table 1:** Communication between PCPs and A/C providers

	PCPs	A/C providers
No effective communication	“We do not get any written documentation of what they’ve done.”	“There’s no effective communication here.”
		“I don’t know if I’m supposed to [communicate with PCPs]. I don’t know if they’re open to it, if they want to hear back how these patients are doing.”
Spotty communication	“I’ve had a few [chiropractors] who have actually have sent me like their note or this improved. And that’s great. That’s wonderful. And I actually wish there was a little bit more of that”	“It’s rare, very rare [to interact with HMO clinician]. Usually, I’m communicating through the client. A couple of times through e-mail”
Contrasting attitudes toward communication	“I think if I were getting reports from acupuncturists, I think that would just annoy me. So I’m kind of glad there’s not a lot of back and forth. I feel like, like getting a report from the dentist, I kind of don’t care.”	“I want to be working in conjunction with a primary care doctor.”
	“Frankly, I don’t have time to call any other providers or anything like that, unless they contact me with a problem. You know, I have way too many things to do…”	“I think it would be wonderful to have an open channel of communication with whatever the doctor is seeing, you know.”
	“I mean, I’m certainly open to it, if someone has something they feel it’s important for me to know. But, the discipline is so very different from Western medicine, that I’m not certain how the information it would provide me would add to what would be familiar enough or make sense to me, to really add anything to what I’m already doing or what I already know.”“I think it [feedback from these providers] would be really helpful. I mean, I think that they probably have insight in terms of the pain…you know, the etiology, the non-physical etiology of the pain.”	“We do our chart notes. And I suppose, yeah, just sending chart notes back and forth. […] all doctors who have a full practice are very busy. And so are acupuncturists. […] would the doctor get the chart note or even want the time to review the chart note. I really don’t know in a perfect world how it could work. But I’m thinking e-mail with just something really quick, back and forth, might reassure the doctor too. I mean, I’m sure doctors worry. What the heck is going on? I haven’t seen this person. And they haven’t been back. And what are they doing? You know, that happens for us too. And we always like when people come back and say, oh, I didn’t keep coming because I got better, so… But there are people that you don’t really know where they stand, you know, how things finished with them or what ended up happening.”“He [PCP] doesn’t know if the patient got better, got worse, who they even went to. I want to use that place again because it seems like they have a pretty good success rate. Doesn’t happen.”

**Table 2:** Communication between patients and PCPs about A/C treatment

	Patients	PCPs
PCP initiated communication	“I had gone to the doctor and he was amazed […] He asked me so many questions in regards to acupuncture and what [the acupuncturist had] done”	“I do follow-ups on the telephone within four to six weeks, or have them follow-up in the office”
Patient initiated communication	“I just always think it’s very good, especially when you’re doing things that are considered complementary or outside their system […] We have to let [doctors] know what we’re doing, what works and what doesn’t work.”	“There’s the outgoing type of patient who’s open to anything. […] they’ll go ahead and describe their experience in great detail, for as long as I’m willing to listen.”
Little communication	“And I don’t volunteer [information]. I mean, I guess I just don’t think of it.”	“It is probably the minority of people that report consistently.”
		“I can’t remember very much in the way of feedback [on acupuncture].”

Given lack of feedback in the referral system, PCPs often did not know whether patients had received or utilized their A/C referrals, where they had gone for treatment, what treatment they received, or what outcomes they experienced. A/C providers on occasion might call a physician or send a note, but this was not systematic. In many cases, A/C providers reached out to PCPs, rather than vice versa. Sometimes those efforts were responded to, and other times not. Some A/C providers were dubious as to the openness or desire of PCPs for communication (see Table 1).

A/C providers expressed openness to providing feedback to PCPs about patient treatment, but were unsure whether such information would be welcomed. Some assumed that physicians were gleaning patient outcomes from direct evaluation of patients and a few encouraged patients to talk to their physicians about positive outcomes. However, patient reports back to PCPs were inconsistent, making many PCPs unaware of outcomes. A/C providers also described how lack of communication of outcomes, particularly positive ones (see Table 3 below about negative bias in outcomes reporting), negatively impacted their ability to demonstrate and advertise their skill and success in treating chronic pain. As one acupuncturist described, more feedback would also ease PCP worries about sending patients out for treatment of which they had no oversight or access.

**Table 3:** Barriers to and challenges for patient-PCP communication

Patients	PCPs
“I guess it just didn’t really cross my mind to discuss it with [my PCP]. I guess he never said, well…Probably if he’d said well, [Name], you know, give me a call or come in and discuss it” “I never did [talk to my PCP about seeing an A/C]. […] I paid for it. They didn’t ask. They didn’t have any interest in any of that.” “[Interaction with PCP has changed] In that, when I go to see my primary care physician, I don’t tell her anything about if I’ve had chiropractic or massage therapy or acupuncture, or anything, because her attitude was not one that seemed like it was…would be received well.” “I just figure that I’ll talk about things that they [PCPs] will help with. And it’s only fifteen minutes. So, I will talk about the other things.” “Fifteen minutes is not enough time when you’re there for a sore throat or for something else. You can’t talk about everything. And I just figure, he really told me he didn’t believe in it. So I just go, forget it. You don’t need to know, I guess.”	“I get no records, and have no chart information, I don’t have anything to look at or review, it’s not like there’s something that’s going to trigger me asking it. Because, you know, if they go see the physical therapist there’s a note. And I can review the note. And I can see that their last three visits were with the therapist. And so I’m much more likely to say, oh, how did it go with the physical therapist? Whereas with the chiropractor, there’s nothing. It’s just a big blank.” “It’s like most of medicine, and what we hear about is failures. Okay? If they get better, nobody bothers calling us back. We only get called back if somebody says, oh, I didn’t get better. Well, if they’re better, they don’t come back and I don’t know.” “If they get the referral, I may not see them again for six months, or nine months, or whatever. And so, no, they don’t make that feedback loop to me that, yeah, it was great benefit. You know, was it the chiropractic, or was it the time that it had actually gotten better. Who knows. But, no, I don’t normally get short term feedback from patients who have gone to chiropractors.”

A number of PCPs indicated that while they might like the system to have more oversight of A/C care, they did not necessarily want, value, or understand the type of treatment feedback from these providers (see Table 1). Coupled with difficulty interpreting A/C notes, time pressures left little time for PCPs to communicate with these providers. Some PCPs also expressed doubt in the veracity of the information, particularly on outcomes, that A/C providers (chiropractors in particular) might claim. In such cases, the patient was the preferred purveyor of treatment information (see below). In rare instances, physicians indicated they would find clinical value in the type of information they might receive from A/C providers. Almost everyone agreed that more communication would be preferable, even, in the case of PCPs, if the information they received from the other provider was not seen as clinically relevant.

The near consensus from both PCPs and A/C providers was there was no effective communication. This, coupled with the negative bias in the occasional reports from patients (see below Table 4), further eroded PCP confidence in these modalities:

“I’ve actually had less happiness with chiropractors the longer I’ve been in practice, just because of what I hear back from patients. […] part of it is I don’t know who I’m referring to. Because it’s this sort of contract, people that we contract with. […] I also don’t get notes back from them. So I have no feedback as to what they’re doing. Whereas, all the Kaiser physical therapists put a note in [the chart]… And I can review what they’ve done, and how many times the patient has gone and the progress they’ve made or not made.”

**Table 4:** Three-way communication issues: A/C provider to patient to PCP

A/C providers	Patients	PCPs
“I tell the patient, you know, your headaches aren’t because of your musculoskeletal system is off or your mechanics is off. Your function is congested. It’s because your pain medication has side effects. So let’s talk about that. And then give them information that they can take back to their primary, and they can change their meds up.”	“Everything is on an electronic record and I’m supposed to get my medical record so I can give it a chiropractor, and then tell my doctor what the chiropractor…It’s like going out on this totally different area. When kind of the allure, at least for me with [HMO], is this kind of big, managed plan. But then I’m encouraged to go off on my own to go do something, without any…You know, it’s not like a chiropractor can look at my MRI. [Someone agreeing] I’m going to have to request my record, you know. And then is my [HMO] provider really going to trust what this chiropractor, who they don’t even know, is going to recommend for my care?”	“Usually what happens is the acupuncturist will tell the patient, this is a weird lump. Get back in to your doctor and have them check it out. And so then they’ll just come back in on their own and say, hey, they told me to come back and get this checked out. I rarely see any…There’s no back and forth otherwise.”
“The patient should always go back to evaluate with their doctors, right? So I would think that the doctor would see the progress, from their patients, their firsthand report. Yeah?”		“And it annoys me when someone comes back with a wrong diagnosis having to do with their leg or their shoulder. It’s completely wrong. And yet they’re like, well, the chiropractor…You know, as if the chiropractor is qualified to diagnose that. […] it irritates me.”
“And, of course, I always tell the patient, especially if they get really good results […] I’ll say, you know, your doc needs to hear about this.”

**Table 5:** Patients as care coordinators

Patients	Pain clinic providers
“I had to be vigilant. And I had to stay on task. And I had to find help […] You know, I have a vested interest in taking care of myself.”	“So when you think about how to integrate the care and how to have it run smoothly, I think that works best if they’re a pretty motivated patient. They can communicate across systems.”
“I’m taking a more active role. I didn’t know, really, what to expect or how to get the train to go the way I wanted it to go, so I kind of let them do the thinking and the planning. And this time, I made it clear from the very first visit that I wanted to look at maintenance.”	“So, for the patient who wants to integrate both into one, I think it then falls on the patient to be carrying […] the information from the acupuncturist to their primary care provider. So it falls on the patient to become that coordinator. And I think, for the most part, patients struggle with that, especially if they’re already dealing with, you know, lots of different health conditions […] it essentially stays un-integrated, unless the patient actively makes that happen.”

### Patient communication with PCP about acupuncture and chiropractic

Given the lack of communication between A/C providers and PCPs, patients are relied upon to ferry information back and forth (see Fig. 1). However, patients varied in the degree to which they discussed their treatments or outcomes with their PCPs. Additionally, in part due to feedback problems in the referral process and access to treatments, PCPs did not have prompts to ask patients about their experience.

As with requesting referral for A/C, patients were often left to initiate discussions about their A/C treatment (see Table 2). While most physicians were not systematic about inquiring about patient experiences, some PCPs asked patients to report back by phone or email after several weeks of A/C. At other times, patients took an active part in ensuring that their PCP was informed of their treatment progress and outcomes. Many patients said it was important that their providers know about the treatments they were using and their experiences with them. This was especially true if an aspect of the treatment might interact with their allopathic treatment, or if they wanted the PCP to know that the treatment was effective so that the PCP would be willing to refer again in the future.

Overall, feedback from patients to physicians was spotty and inconsistent. PCPs and patients described several issues that interfere with patient feedback to PCPs about A/C treatment (see Table 3). Physicians especially noted the problem of negative bias in reporting, which can skew their overall impression of the effectiveness of A/C treatment. PCPs said if patients return for another appointment soon after referral, it is often because the treatment was ineffective, or for another purpose and thus the A/C treatment does not come up. Indeed, patients expressed that because of time barriers, they would selectively utilize doctors’ office visits to bring up issues of current import. For those with successful reduction in pain, too, it might be months before they are back to see the PCP, and several PCPs noted that this would cause them to question whether time or the treatment had caused the improvement. In addition, because doctors do not receive direct feedback about referrals, unless the patient mentions treatment, there is no prompt to facilitate the PCP inquiries.

Patients described a number of reasons why they might not initiate discussions about their treatment (see Table 3). Many said that the degree to which they discussed their A/C treatment depended, in part, on their physicians’ receptivity and understanding of it. Other times, patients did not think the treatment information was relevant to their allopathic doctors. Many noted that because their PCP had never asked them about it, they never volunteered the information.

Discrepancies between allopathic and CAM explanatory models, transmitted via patients, were described as causing problems for PCPs in particular. Patients would sometimes be sent back to PCPs with new diagnoses or requests for additional investigation that did not always make sense from a biomedical model (see Table 4). Some patients expressed concern about the lack of communication between their PCPs and A/C providers, and articulated desires that there be more communication between them. Barriers to communication between providers placed patients, often uncomfortably, in the middle.

### Patients as care managers

While both HMO providers and A/C providers continually pointed to and deferred to PCPs as the responsible party in patient care, it was implicit, and sometimes explicit, that ultimate responsibility for accessing, coordinating, and managing care fell on the patient (see Table 5). This is particularly the case when patients are using allopathic and CAM treatments, and providers both inside and outside the HMO.

As the above discussions illustrate, the lack of feedback and communication results in patients being charged with channeling communications that are using different languages and explanatory models, which are not mutually intelligible. Often CAM providers use the same words as PCPs, but the meaning and intentions behind the words are different. Patients must work very hard to use conventional and CAM therapies. Patients are aware that in order to have their needs met, they have to work the system to access care. As one patient reflected, “I had to be vigilant. And I had to stay on task. And I had to find help […] You know, I have a vested interest in taking care of myself.” However, not all patients have the knowledge of the system, or the capacities and resources necessary to communicate across it, to hold everything together and access available care.

## Discussion

Our analyses of the main players in the CAM health care triad highlight deficiencies of communication between PCPs and A/C providers, with patients being left to manage the information and communication. In line with previous research [16, 17, 28], we found that the two clinicians, PCP and A/C, manage the patient not as a team, but in parallel. They do not have a relationship with one another, so there is no basis for communication and mutual understanding. Indeed, there was very little person-to-person exchange of information from A/C providers to PCPs, and essentially none from PCPs to A/C providers. As a result, there is little learning that takes place for either type of practitioner. As noted above, feedback would be mutually beneficial to PCPs and A/C providers, as it might be able to facilitate referrals to proven, successful providers.

In these respects the issues that are highlighted may be similar to some deficiencies in communication between PCPs and subspecialists [34, 35]. Within the HMO, there are the usual care integration problems, such as practitioners not fully reviewing incoming charts and care being provisioned at different sites. However, in comparison to CAM treatments, allopathic care was discussed by our participants as integrated through the patient medical record and through the housing of providers within HMO facilities. In addition to the HMO providing structure for integration, allopathic care was integrated through documenting treatment in a language based on common assumptions about human physiology and pathology. In the case of CAM, the two clinicians, PCP and CAM provider, may be operating from different paradigms, with different explanatory models, different diagnoses, and different expectations for outcomes (see also [21, 36, 37]), a concern expressed more by the PCPs above than by the A/C providers. In our setting, while the A/C providers were reimbursed by the HMO, their practices were largely outside the HMO network. They maintained separate patient medical records from the PCPs, and could not easily provide patient updates to the patient’s HMO medical record. While the A/C providers expressed a desire to share information, the PCPs were skeptical of the potential clinical value of such sharing, even while complaining that they did not know about how their patients were being treated and wanted more information about it.

The only communication bridge between these parallel worlds is the patient. Often the patient’s first task is to raise the issue of A/C treatment in order to obtain a referral, a conversation that may be a difficult one to initiate. Once referred and receiving A/C treatment, all communication between practitioners has to occur via the patient, who attempts to ferry critical information back and forth (for similar examples from breast cancer and gynecology see [38, 39]). This requires that a successful patient be resourceful, savvy, and persistent. On top of that, from the perspective of both allopathic and CAM providers, patients must “do their part” by actively engaging in self-care. Programs such as the pain management group provide training ground for patients to learn about care options and make, apparently, informed decisions about treatment. These groups, along with self care advice given by all providers, educate patients in order to empower them and make them invested and “active” parties to their treatment. It also reinforces the construction of patient as both treatment coordinator and care manager. However the task is far too complex for most patients to have much chance of success when they are not sure what their roles are or the PCP reception of what they have to say, and when they might not share any or all of the information related to their CAM treatment [24, 25, 33, 40, 41].

The current study is limited by several methodological factors. First, the study was conducted within an HMO and many of the communication issues, especially between A/C and allopathic providers, were influenced by particularities of the HMO’s structure and processes. This possibly limits the generalizability of our findings. Future research should examine the triad in other managed care or non-managed care settings. Second, we rely on patient and provider self-report. We were unable to observe and document actual interactions between members of the triad. Future research might incorporate an observational component of patient office visits, as well as examine written communications, to study first-hand the communicative exchanges. Third, our community A/C provider sample size was narrow because of pragmatic recruitment considerations. This was not a free-standing qualitative study, but rather embedded as Phase 2 in a larger mixed-methods study of outcomes associated with acupuncture and chiropractic care (see [32] for an overview of all components of the study.) Additional research might broaden the sample to include providers receiving some, but not a lot of referrals from the HMO to see how their experiences differ or are similar to those with high rates of referrals. Finally, in our analyses we considered acupuncturists and chiropractors as a single group of clinicians. We took this approach as representing the vantage point of the managed care network and the primary care physicians who, from the standpoint of policy and clinical integration, may likely view interactions these 2 groups as raising similar categories of issues. Future research might focus on exploring the distinctions between acupuncture and chiropractic clinicians in terms of their relationships with the conventional healthcare providers.

## Conclusions

The communication hiatus identified in our research may be viewed as a major contributing factor to the ongoing chronic pain management/chronic opioid therapy conundrum. CAM plays a major role in the management of chronic pain for many patients [6]. Thus the inefficiencies and quality of care deficiencies inherent in such a dysfunctional communication system may be contributing materially to suboptimal outcomes. Improvements in PCP/CAM provider communication could contribute to improved care for individual patients, and improved patient management algorithms with properly coordinated care. How might such improvements in communication be achieved? One important step wherever feasible would be to include progress notes from CAM visits in the electronic medical record, while likewise providing some type of access to the electronic medical record to CAM practitioners (cf. [42, 43]), such as would occur if the health plan included A/C on staff. This would allow for at least some exchange of clinical data. On the other hand, we know that PCPs and subspecialists share the same electronic medical record access, and between those two groups many of the same communication challenges exist. In any case, further research and policy initiatives are needed to delineate mechanisms for improving communication and understanding among the various classes of clinicians caring for patients with chronic pain. Finally, the system does not make explicit to patients their important role in communication between providers. Short of other solutions, it may be reasonable to identify strategies for more clearly empowering patients to step into the void.

Finally, the results of the study may be viewed as strongly arguing for the use of integrative medicine clinicians within established biomedical health system, as a mechanism for providing, and integrating this type of care. However, such a strategy cannot stand alone at this time, because it cannot be fully scaled. That is to say, the number of integrative medicine practitioners is still relatively small, while the volume of acupuncture and chiropractic use is high.

## Abbreviations

A/C: acupuncture and chiropractic
CAM: complementary and alternative medicine
CMP: chronic musculoskeletal pain
HMO: health maintenance organization
PCP: primary care physician
